# Multiplex RT-qPCR strategy for SARS-CoV-2 variants detection in developing countries without ngs: The Bolivian experience

**DOI:** 10.1017/S095026882510037X

**Published:** 2025-08-15

**Authors:** Rudy Parrado, Carolina X. Cuba-Grandy, Eugenia Fuentes-Luppichini, Nattaly Grecia Torrico Villarroel, Yercin Mamani-Ortiz, Jaqueline Mendez, Betty Melgarejo, Irenice Coronado-Arrázola, Nair A. Montaño, Leonardo I. Almonacid, Rafael A. Medina, Lineth Garcia, Catalina Pardo-Roa

**Affiliations:** 1 Instituto de Investigaciones Biomédicas (IIBISMED), Universidad Mayor de San Simón, Cochabamba, Bolivia; 2 Facultad de Ciencias Farmacéuticas y Bioquímicas, Universidad Mayor de San Simón, Cochabamba, Bolivia; 3 Department of Pediatric Infectious Diseases and Immunology, School of Medicine, Pontificia Universidad Católica de Chile, Santiago, Chile; 4 Departmental Health Service of Cochabamba, Cochabamba, Bolivia; 5 Molecular Bioinformatics Laboratory, Department of Molecular Genetics and Microbiology, Faculty of Biological Sciences, Pontificia Universidad Católica de Chile, Santiago, Chile; 6 School of Engineering, Medicine and Biological Sciences, Institute for Biological and Medical Engineering, Pontificia Universidad Católica de Chile, Santiago, Chile; 7 Emory Center of Excellence of Influenza Research and Response (Emory-CEIRR), Atlanta, USA; 8 Center for Research on Influenza Pathogenesis and Transmission (CRIPT) Center of Excellence of Influenza Research and Response (CEIRR), New York, USA; 9 Department of Pathology and Laboratory Medicine, School of Medicine, Emory Vaccine Center, Emory University, Atlanta, USA; 10 Department of Child and Adolescent Health, School of Nursing, Pontificia Universidad Católica de Chile, Santiago, Chile

## Abstract

The rapid evolution of SARS-CoV-2 has led to the emergence of variants of concern (VOCs) characterized by increased transmissibility, pathogenicity, and resistance to neutralizing antibodies. Identifying these variants is essential for guiding public health efforts to control COVID-19. Although whole genome sequencing (WGS) is the gold standard for variant identification, its implementation is often limited in developing countries due to resource constraints. In Bolivia, genomic surveillance is a challenge due to its limited technological infrastructure and resources. An RT-qPCR-based strategy was designed to address these limitations and detect the mutations associated with VOCs and variants of interest (VOIs). The multiplex RT-qPCR commercial kits Allplex^TM^ Master and Variants I (Seegene®) and the ValuPanel^TM^ (Biosearch®) were used to target mutations such as HV69/70del, E484K, N501Y, P681H, and K417N/T. They are characteristic of the Alpha (B.1.1.7), Beta (B.1.531), Gamma (P.1), Omicron (B.1.1.529), Mu (B.1.621), and Zeta (P.2) variants. A total of 157 samples collected in Cochabamba from January to November 2021 were evaluated, identifying 44 Gamma, 2 Zeta, 20 Mu, and 10 Omicron were identified. The strategy’s effectiveness was validated against WGS data generated with Oxford Nanopore^TM^ technology, showing a concordance rate of 0.96. This highlights the value of the RT-qPCR strategy in guiding the selection of samples for WGS, enabling broader detection of new variants that cannot be identified by RT-qPCR alone.

## Short report

The global pandemic for COVID-19, officially declared between March 2020 and May 2023, was caused by the Syndrome Acute Respiratory Severe coronavirus type 2 (SARS-CoV-2). It had significantly affected over 200 countries and territories, resulting in more than 766 million confirmed cases and nearly 7 million reported deaths. Since the virus was identified in December 2019 in Wuhan, China, the World Health Organization (WHO) has highlighted the importance of genomic sequencing in understanding viral evolution, developing vaccines, optimizing diagnostic tools, and refining public health strategies [1].

In December 2020, distinct variants emerged, exhibiting evolutionary advantages such as increased transmissibility and disease severity, reduced neutralization by the immune response, and compromised efficacy of treatments, vaccines, and diagnostic tests [2]. In 2021, the WHO established a consensus nomenclature to classify these emerging lineages based on their epidemiological and clinical impact, designating them as Variants of Concern (VOCs), Variants of Interest (VOIs), and Variants Under Monitoring (VUMs) [3]. The first VOCs to emerge were Alpha (B.1.1.7), Beta (B.1.531), Gamma (P.1), Delta (B.1.617), and Omicron (B.1.1.529), while VOIs such as Mu (B.1.621), Lambda (C.37), and Zeta (P.2) were also identified during this period [1].

The characteristics of these variants are conferred by distinct mutations mainly located in the receptor-binding domain (RBD) of the Spike (S) protein gene, which mediates the virus’s affinity for the host cell receptor ACE2 (angiotensin-converting enzyme 2). Mutations related to emerging variants include N501Y and HV 69/70del, both reported in the Alpha variant (B.1.1.7) and associated with enhanced transmissibility. The E484K mutation, present in Beta (B.1.531) and Gamma (P.1) variants, reduces the virus’s susceptibility to neutralization by antibodies, compromising immune protection [2, 3].

The timely identification of these variants was critical to monitor and control viral spread. In developing countries like Bolivia, where genomic sequencing capabilities were severely limited, public health decisions were made based on regional contexts rather than national data. In January 2021, a year into the pandemic, 27 sequences from Bolivia were shared for the first time, all of which had been sequenced abroad. In some cases, the official reports were delayed by up to three months between sample collection and official reporting, resulting in a substantial delay for local decision-makers in preventing and controlling the spread of new variants. By July 2021, the global transmission of VOCs was widespread, and new variants continued to emerge. In this context, nearly 2 million genomic sequences were shared on the GISAID platform. However, although Bolivia recorded approximately 460,000 COVID-19 cases during the study time, only 66 sequences were submitted to international databases (≈0.01%) [3]. Notably, 56 of Bolivia’s sequences were processed outside the country by FIOCRUZ (Brazil), with some uploaded to the platform as late as a year after the biological samples were collected. By the end of the year, 155 sequences were shared, of which only 7% were sequenced in Bolivia. The lack of local sequencing technology for rapid genomic surveillance limited Bolivia’s ability to understand the real-time dynamics of viral spread fully, consequently delaying critical public health decision-making to control the virus’s spread [4, 5].

Although whole genome sequencing (WGS) is the most accurate tool for tracking mutations, its high costs and infrastructure demands make it less feasible in countries where resources are prioritized for diagnostics and treatment. Reverse Transcriptase Quantitative Polymerase Chain Reaction (RT-qPCR) represents a more accessible and easier alternative implementation. In addition, it has been widely used for COVID-19 diagnosis, and specific protocols have been developed to identify mutations associated with VOCs and VOIs. Strategically employing these kits offers a valuable approach for molecular-level epidemiological surveillance, offering results comparable to genomic sequencing at a significantly reduced operational cost [6–8].

This study describes the development and implementation of an RT-qPCR strategy for SARS-CoV-2 variant surveillance in Cochabamba, Bolivia, using commercially available kits at the time of this study in 2021: RT-qPCR Allplex™ SARS-CoV-2 Master Kit (Seegene Technology), Allplex™ SARS-CoV-2 Variants I kit (Seegene Technology) and Variant Value Panel (Biosearch Technology). These assays were designed for research use only and did not have IVD (In vitro Diagnostic) registration; they are no longer commercially available. The strategy was designed to initially identify the VOCs Alpha (B.1.1.7), Beta (B.1.531), Gamma (P.1), and later adapt the strategy to detect the VOC Omicron (B.1.1.529) and the VOIs Zeta (P.2) and Mu (B.1.621).

The RT-qPCR strategy was applied to 145 nasopharyngeal swab samples collected between January and June 2021 from patients aged 18 to 59 years diagnosed with COVID-19 by RT-qPCR with cycle threshold (Ct) values below 30. Samples were randomly selected based on epidemiological criteria, respecting the proportional distribution of cases identified in the five geographic regions of the Department of Cochabamba and the percentage of cases by month in every region. Additionally, given the regional epidemiological situation, where the Omicron variant was already widely circulating, 12 additional samples collected between August and October 2021 were included in the study to set this strategy for detecting this variant. In total, 157 samples were analyzed. This study followed the ethics guidelines of the Bioethics Committee of the Faculty of Medicine, Universidad Mayor de San Simón under protocol No. CE-13.

The nasopharyngeal swab was resuspended in a universal transport medium, and RNA was extracted using the QIAamp Viral RNA Kit (QIAGEN, Cat. No. 52904) according to the manufacturer’s instructions, yielding 70μLof eluted viral RNA. RT-qPCR diagnosis was performed using the Bio-Rad CFX96 qPCR system with the genome detection panel from the CDC (USA) and the iTaq™ Universal Probes One-Step Kit (Bio-Rad).

The RT-qPCR strategy involved a sequential approach, as described in Table 1. First, Allplex™ SARS-CoV-2 Master Kit (Seegene) was used to screen for mutations in the spike gene (HV69/70 deletion, Y144 deletion, E484K, N501Y) in a single reaction without individual differentiation, distinguishing *mutant strains* from non-mutated strains, referred to as *No-VOCs* in this study. Samples with detected spike gene mutations were then analyzed using the Allplex™ SARS-CoV-2 Variants I kit (Seegene Technology), which detected the HV69/70 deletion, E484K, and N501Y mutations in a single reaction. Results from both kits were analyzed using Seegene Viewer V1.0 (Seegene) to provide a preliminary classification of Alpha, Gamma, and Beta variants. Finally, the Variant Value Panel (Biosearch Technology) was used to identify the K417N, K417T, and P618H mutations in separate reactions, with results interpreted through allele discrimination plots. Table 1 summarizes the mutations in the S gene associated with the VOCs Alpha (B.1.1.7), Beta (B.1.531), Gamma (P.1), Omicron (B.1.1.529), and the VOIs Zeta (P.2) and Mu (B.1.621), distinguishing them from one another. All three RT-qPCR kits were processed using the Bio-Rad CFX96 qPCR system.

**Table 1.** Summary of result interpretation for variants using RT-qPCR strategy

TEP 1: *Mutations identified from positive samples of the S protein gene, mutated by the Allplex™ SARS-CoV-2 Master Kit (Seegene)*.						INTERPRETATION WHO Variant (Pango Lineage)	Concordance Between RT-qPCR Strategy and Nanopore Sequencing for SARS-CoV-2 Variants		Key Mutations in the SARS-CoV-2 S Gene Related to Variants (2)
STEP 2: *Allplex™ SARS-CoV-2 Variants I kit (Seegene)*			STEP 3: *SARS-CoV-2 Variant Value Panel (Biosearch Technology)*				Variant by RT-qPCR N^0^/Total (%)	Variant by Nanopore WGS N^0^/Total (%)	
N501Y	HV69/70 del	E484K	K417T	K417N	P618H				
+	+				+	VOC: Alpha (B.1.1.7)	0 (0%)	0 (0%)	**HV69/70del, N501Y, P618H,** Y144del, P618H, K1191N, L5F, S98F, W152R, D138H, L18F, I68del, T572I
+		+		+		VOC: Beta (B.1.531)	0 (0%)	0 (0%)	L18F, **K417T, E484K, N501Y**, D614G, A27S, T19I, P384L, T240del, A899S, L5F, A67V, S689R, Y144del
+		+	+			VOC: Gamma (P.1)	44/44 (100.0%)	6/6 (100.0%)	L18F, T20N, P26S, D138Y, R190S, **K417T, E484K, N501Y**, D614G, H655Y, T1027I, V1176F
		+				VOI: Zeta (P.2)	2/2 (100.0%)	2/2 (100.0%)	**E484K,** G614G, Q677H, V1176F
+		+			+	VOI: Mu (B.1.621)	20/20 (100.0%)	9/9 (100.0%)	T95I, Y144S, Y145N, R346K, **E484K, N501Y**, D614G**, P618H,** D950N
+	+			+	+	VOC: Omicron (B.1.1.529)	10/10 (100.0%)	10/10 (100.0%)	**HV69/70del, N501Y, P618H,** D614G, E484A, Y144del, **K417N**, T478K, H655Y, N764K, Q493R, D796Y, N679K, Y505H
Mutations were identified from negative samples of the S protein gene, mutated by the Allplex™ SARS-CoV–2 Master Kit (Seegene).						*non-VOC* Strain: Candidate to WGS	80/81 (98.8%)	13/14 (92.9%)	-
TOTAL							156/157*	40/41**	

* One of the analyzed samples tested positive during screening with the Allplex™ Master (Seegene Technology) but did not exhibit any specific mutations analyzed by the RT-qPCR strategy. Consequently, this sample could not be classified using the current methodology.

** Another sample categorized as a *non-VOC* strain by the RT-qPCR strategy was identified as the Lambda variant (a variant of interest) through sequencing. The mutations associated with the Lambda variant are not covered by the multiplex RT-qPCR kits utilised.

A subset of 41 samples (28.28%) was randomly selected to validate the RT-qPCR strategy for whole-genome sequencing (WGS) at the Molecular Virology Laboratory of the Pontificia Universidad Católica de Chile, using the ARTIC SARS-CoV-2 protocol on a MinION™ Mk1C sequencer (Oxford Nanopore Technologies). Briefly, cDNA synthesis and ARTIC V3/V4 multiplex PCR were performed as described by Tyson *et al*. [9] and sequencing libraries were prepared according to the manufacturer’s instructions for Native Barcoding kit 96 V14, and genomes were assembly as described previously [10]. SARS-CoV-2 clades and lineages were assigned employing Nextstrain and Pangolin nomenclatures. Complete genomes (>95% coverage) were uploaded to the GISAID platform. Derived data supporting the findings of this study are available from the corresponding author, CPR, on request.

The concordance between the RT-qPCR strategy and genomic sequencing was 97.5%, demonstrating adaptability to emerging variants. The strategy successfully classified biological samples as Alpha (B.1.1.7), Beta (B.1.531), Gamma (P.1), Omicron (B.1.1.529), Zeta (P.2), and Mu (B.1.621) variants, focusing on the identification of key mutations: N501Y, E484K, K417N/T, HV69/70del, and P618H.

The results show that, in 2021, a total of 157 samples were analyzed, identifying 44 Gamma (P.1), 2 Zeta (P.2), 20 Mu (B.1.621), and 10 Omicron variants. Additionally, 81 samples were classified as non-VOC, meaning they did not carry mutations of interest. Among 41 samples subjected to WGS, the distribution was as follows: 6 Gamma, 2 Zeta, 9 Mu, and 10 Omicron. These results are summarized in Table 1.

Genomic surveillance of SARS-CoV-2 was conducted using the strategy designed with the three RT-qPCR kits with a total of 145 nasopharyngeal swab samples collected between January and June 2021 in Cochabamba. The results show circulation of the Zeta (P.2) variant first, followed by Gamma (P.1), and then Mu (B.1.526). By June 2021, Gamma and Mu were competing for predominance (Supplementary Figure S1). All three variants circulated in the Metropolitan region. In contrast, the *non-VOC strain* predominated in the Andean region, and Gamma (P.1) was predominant in the Tropical and Southern Cone regions (Supplementary Figure S2).

When comparing the results obtained from genomic surveillance, no agreement was found with reports from health authorities in the Cochabamba department during the first half of 2021. According to the information released by the Ministry of Health of Bolivia, no VOCs or VOIs were identified as circulating in the department until May 2021 [5]. Furthermore, on the GISAID platform, there were only six shared sequences representing the Cochabamba’s Department [4]. These data suggest a lack of attention to genomic surveillance in this department and impacted the delay in implementing preventive and control measures by health decision-makers at the local and national levels. To our knowledge, no studies have been conducted on the spatial spread of specific variants in other departments of Bolivia using PCR techniques, making this work the first report on SARS-CoV-2 surveillance using RT-qPCR in the country.

This study highlights the importance of adapting genomic surveillance methodologies to local resources in countries without access to massive sequencing technology and particularly in low-resource settings, to ensure a quick and effective response to the emergence of infectious outbreaks. The experience in Bolivia exemplifies this, where emerging SARS-CoV-2 variants were successfully identified despite the absence of whole genome sequencing (WGS) capabilities and through RT-qPCR-based surveillance. This approach demonstrates that timely detection and monitoring of viral variants are possible even without massive sequencing infrastructure, which is essential for guiding public health interventions during epidemics.

The designed RT-qPCR multiplex strategy enables laboratories without sequencing capabilities to identify variants of concern and interest, aiding in epidemiological research, diagnostics, and pandemic control. It also facilitates large-scale surveillance and sample screening for WGS analysis. Therefore, its use can be promoted in local laboratories within the public health system in low- and middle-income countries, facilitating access to real-time information for decision-making and the implementation of local health policies in cases of the emergence of infectious outbreaks.

It is important to note that commercial RT-qPCR kits are designed according to the epidemiological context of their countries of origin (the USA and Europe), which can limit their ability to detect variants prevalent in Latin America [7]. For example, one sample initially classified as *non-VOC* by this strategy was later identified as Lambda (C.37) via WGS, highlighting the need for complementary screening approaches with the RT-qPCR strategy.

The future perspective of this methodology aims to continuously adapt by developing new primers and probes targeting emerging mutations. While qPCR enables rapid detection of known variants, its key value lies in screening samples that remain unclassified, which can then be prioritized for WGS. This targeted sequencing optimizes resource use by focusing on samples most likely to reveal novel variants. With commercial kits no longer available, the emphasis now shifts toward developing in-house protocols tailored to regional epidemiological realities. These in-house strategies are not only essential for sustaining genomic surveillance in the absence of commercial kits but also represent a first step toward long-term diagnostic autonomy. Following their successful application during emergencies such as the COVID-19 pandemic, these protocols should aim to transition from research-use-only tools to registered in vitro Diagnostic (IVD) assays. This would ensure their validation, standardization, and broader use in routine diagnostic workflows, reinforcing preparedness for future outbreaks.

Ultimately, the goal is to establish flexible, accessible qPCR methods as frontline tools for SARS-CoV-2 genomic surveillance, particularly in settings lacking widespread WGS access. This strategy empowers laboratories in resource-limited countries to take control of their genomic surveillance efforts, overcoming technological barriers and enabling timely, accurate detection of emerging variants through innovative, resource-efficient approaches.

## Supporting information

10.1017/S095026882510037X.sm001 Parrado et al. supplementary material Parrado et al. supplementary material

## Data Availability

The data that support the findings of this study are available at GISAID database under the access IDs EPI_ISL_15184051 to EPI_ISL_15184056; EPI_ISL_16765648 to EPI_ISL_16765663, EPI_ISL_17987272, EPI_ISL_17987273, EPI_ISL_17987273, EPI_ISL_18437742 to EPI_ISL_18437758, EPI_ISL_19391227 to EPI_ISL_19391253, EPI_ISL_19601692 to EPI_ISL_19601753 and EPI_ISL_19601809 to EPI_ISL_19601812. Additional data supporting the findings of this study are available from the corresponding author, CPR under request.

## Supplementary material

The supplementary material for this article can be found at http://doi.org/10.1017/S095026882510037X.
