# Supplementary material for: Multiplex RT-qPCR strategy for SARS-CoV-2 variants detection in developing countries without ngs: The Bolivian experience
Source: Epidemiol Infect. 2025 Aug 15;153:e94. doi: 10.1017/S095026882510037X (PMC12394009; doi:10.1017/S095026882510037X)
Supplement: Parrado et al. supplementary material [file S095026882510037Xsup001.docx]

**Supplementary material**


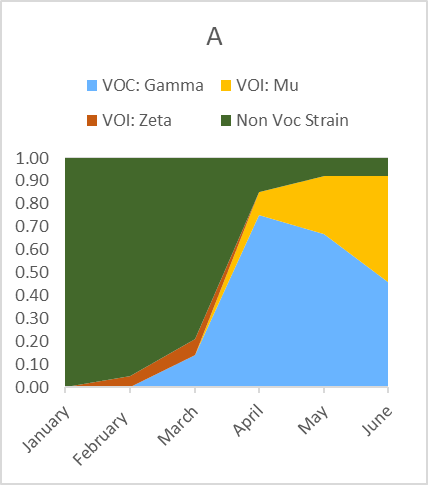

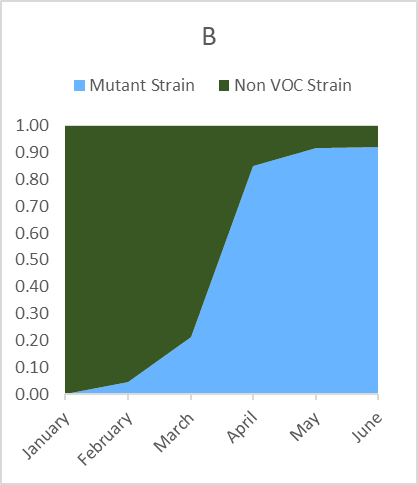


**Supplementary Figure 1. Temporal Dynamics of Circulating Variants in Cochabamba, Bolivia.** A) Illustrates the emergence and increase in circulation of VOCs/VOIs during the study period. B) Shows the shift in predominance between variants with mutations related to VOCs/VOIs (N501Y, E484K, K417N/T, HV69/70del, P618H) and non-VOC strains (viruses lacking these mutations) between March and April 2021.


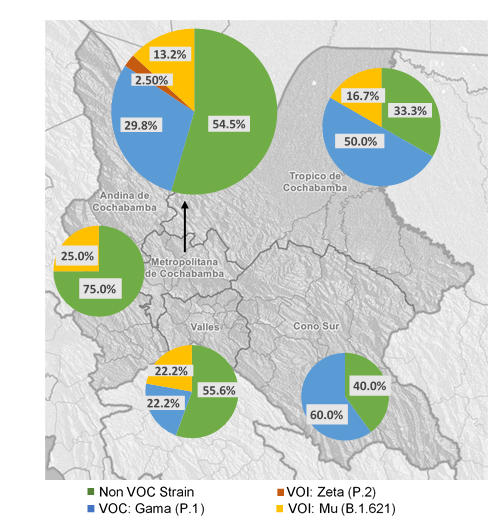


**Supplementary Figure 2.** Geographic Distribution of Variants in the Five Geographic Regions of Cochabamba, Bolivia.
